# Editorial: Precision medicine approaches in radiotherapy and systemic therapy of brain metastases

**DOI:** 10.3389/fonc.2023.1190830

**Published:** 2023-04-20

**Authors:** David Kaul, Anna S. Berghoff, Matthias Guckenberger

**Affiliations:** ^1^ Department of Radiation Oncology and Radiotherapy, Charité-Universitätsmedizin, Freie Universität Berlin, Humboldt-Universität zu Berlin and Berlin Institute of Health, Berlin, Germany; ^2^ Department of Medicine 1 and Comprehensive Cancer Center Vienna , Medical University of Vienna, Vienna, Austria; ^3^ Department of Radiation Oncology, University Hospital Zurich, University of Zurich, Zürich, Switzerland

**Keywords:** precision medicine, brain metastases, immunotherapy, targeted therapy, radiotherapy

The incidence of brain metastases is on the rise, partly due to improved imaging techniques that enable more accurate detection and partly because of increased survival rates in cancer patients due to improvements in systemic therapy. As cancer care evolves, it becomes increasingly important to explore and develop novel approaches to manage brain metastases, ensuring that patients receive the most effective treatments tailored to their individual needs. In this special edition, we present nine comprehensive articles that delve into the promising and rapidly evolving field of precision medicine in the context of radiotherapy and systemic therapy of brain metastases. The continuing development and application of advanced technologies and therapies are reshaping the landscape of cancer treatment.

In the article, “*Radiosurgery for Five to Fifteen Brain Metastases: A Single Centre Experience and a Review of the Literature*” by Rogers et al. the authors examine the clinical outcomes of patients with five or more brain metastases treated with stereotactic radiosurgery (SRS). The study highlights excellent local control rates and demonstrates that overall survival following SRS for multiple brain metastases is determined by the course of the extracranial disease. This article contributes to our understanding of SRS’s potential and limitations in treating carefully selected patients with multiple brain metastases.

In the article, “*Radiomic Signatures for Predicting Receptor Status in Breast Cancer Brain Metastases*,” Luo et al. examine receptor discordance between primary breast cancers and brain metastases. They establish radiomic signatures using preoperative brain MRI to predict receptor status (estrogen receptor, progesterone receptor, and human epidermal growth factor receptor 2) in metastases. The study concludes that receptor conversion is common, and radiomic signatures show potential for noninvasively predicting receptor status, which could inform therapeutic decisions.

In the article, “*Current Treatment Approaches and Global Consensus Guidelines for Brain Metastases in Melanoma*,” Tan et al. review global consensus guidelines for treating melanoma brain metastases (MBM),. These guidelines provide valuable guidance for clinical decision-making in MBM treatment.

The “*Systematic literature review and meta-analysis of clinical outcomes and prognostic factors for melanoma brain metastases*” presents a systematic review and meta-analysis of clinical outcomes and prognostic factors in melanoma brain metastases (MBM) patients (Tan et al.). The analysis included 41 observational studies and 12 clinical trials on treatment outcomes, as well as 31 observational studies on prognostic factors. This study provides valuable insights into the association between patient characteristics and MBM prognosis, helping guide clinical decision-making.

In the article, “*Predictors of Lung Adenocarcinoma With Leptomeningeal Metastases: A 2022 Targeted-Therapy-Assisted molGPA Model*,” Zhang et al. explore prognostic indicators of lung adenocarcinoma with leptomeningeal metastases (LM) and provide an updated graded prognostic assessment model integrated with molecular alterations (molGPA). The 2022 molGPA model demonstrates better prognostic performance than previous models, making it useful for clinical decision-making and stratification in future clinical trials

In the article, “*Radiomic Signatures for Predicting EGFR Mutation Status in Lung Cancer Brain Metastases*,” the authors create a radiomic model using preoperative brain MR images from 162 patients (Zheng et al.). The best-performing model demonstrates high classification accuracy, sensitivity, and specificity. The study concludes that radiomic signatures can potentially noninvasively predict the EGFR mutation status of lung cancer brain metastases, impacting prognosis and treatment decisions.

The article, “*Clinical determinants impacting overall survival of patients with operable brain metastases from non-small cell lung cancer*,” aims to improve clinical decision-making by investigating factors affecting survival in patients with resectable NSCLC brain metastases (Piffko et al.). A retrospective analysis was conducted on 264 patients, which identified several factors that impacted overall survival, such as the systemic metastatic load and the number of brain metastases (solitary vs. singular and multiple BM). The study also identified age, Karnofsky Performance Status, and gender as factors impacting survival. These findings contribute to a better understanding of the risks and course of the disease, ultimately aiding clinical decision-making in tumor boards.

The article “*The value of stereotactic biopsy of primary and recurrent brain metastases in the era of precision medicine*” investigates the diagnostic yield and safety of image-guided frame-based stereotactic biopsy (STX) in brain metastases patients (Katzendobler et al.). The retrospective study found that STX provided a definitive diagnosis in 98% of cases, with a 95% success rate in molecular genetic analyses. The procedure had a low complication rate of 2.4%, with no permanent morbidity or mortality. This study highlights STX’s potential to enable precision medicine approaches in treating primary and recurrent brain metastases.

Finally, in “*What if: A Retrospective Reconstruction of Resection Cavity Stereotactic Radiosurgery to Mimic Neoadjuvant Stereotactic Radiosurgery*” examines neoadjuvant stereotactic radiosurgery (NaSRS) of brain metastases and its impact on normal brain tissue (NBT) (Acker et al.). The study analyzed hypothetical pre- and actual postoperative target volumes in 30 patients, finding that smaller tumors had a higher risk of volume increase when irradiated postoperatively. Precise delineation is crucial, as it directly affects NBT exposure, but contouring resection cavities is challenging. The article highlights the need for further research to identify patients at risk for significant volume increase, who may benefit from NaSRS. Ongoing clinical trials will further evaluate the benefits of this approach.

Collectively, these nine articles emphasize the potential of precision medicine approaches in radiotherapy and systemic therapy of brain metastases. We are moving closer to a future where personalized cancer treatment is the norm. We hope that the insights presented in this special Research Topic will inspire further research and innovation in this critical field. As the incidence of brain metastases continues to rise, it is of utmost importance that we continue to explore new treatment options and refine existing techniques to provide the best possible care for patients affected by this challenging condition. By fostering collaboration among researchers, clinicians, and the broader scientific community, we can work together to make significant strides in our ongoing battle against cancer.

## Author contributions

All authors listed have made a substantial, direct, and intellectual contribution to the work and approved it for publication.

